# Supplementary material for: An open-source framework for large-scale, flexible evaluation of biomedical text mining systems
Source: J Biomed Discov Collab. 2008 Jan 29;3:1. doi: 10.1186/1747-5333-3-1 (PMC2276192; doi:10.1186/1747-5333-3-1)
Supplement: Additional file 1 — Evaluation platform source code. The additional file contains the software evaluation framework discussed in this paper. The most current release can be downloaded from SourceForge.net [1]. See the accompanying README file for instructions on installation and use. Also included in the distribution is a collection of UIMA wrappers for some commonly used BioNLP tools and annotated corpora. [file 1747-5333-3-1-S1.zip › bionlp_uima_repository-1.0.1/docs/api/src-html/edu/uchsc/ccp/util/nlp/parser/corpus/TexasCorpusParser.html]

```
001    package edu.uchsc.ccp.util.nlp.parser.corpus;
002    
003    import java.io.BufferedReader;
004    import java.io.File;
005    import java.io.IOException;
006    import java.util.ArrayList;
007    import java.util.List;
008    import java.util.Stack;
009    import java.util.regex.Matcher;
010    import java.util.regex.Pattern;
011    
012    import edu.uchsc.ccp.util.nlp.GenericTextUtils;
013    import edu.uchsc.ccp.util.nlp.annotation.AnnotationSet;
014    import edu.uchsc.ccp.util.nlp.annotation.Annotator;
015    import edu.uchsc.ccp.util.nlp.annotation.TextAnnotation;
016    import edu.uchsc.ccp.util.nlp.document.GenericDocument;
017    import edu.uchsc.ccp.util.nlp.mention.ClassMentionTypes;
018    
019    /**
020     * A parser for the Texas corpus. This class outputs the original text and accompanying annotations for each Texas corpus document. Titles and
021     * abstracts are outputted as separate documents.<br>
022     * <br>
023     * Output Classes:<br>
024     * <ul>
025     * <li>protein</li>
026     * </ul>
027     * 
028     * @author williamb
029     * 
030     */
031    public class TexasCorpusParser extends CorpusParser {
032        public final static int DOCUMENT_COLLECTION_ID = 12;
033    
034        private static List<TextAnnotation> allAnnotations = new ArrayList<TextAnnotation>();
035    
036        private static List<String[]> allDocuments = new ArrayList<String[]>();
037    
038        private static final String PROTEIN_TAG = "prot";
039    
040        private final static boolean DEBUG = false;
041    
042        private static Stack<TextAnnotation> openAnnotationStack;
043    
044        private final static Annotator annotator = new Annotator(new Integer(13), "", "Texas", "UTCS");
045    
046        private final static AnnotationSet annotationSet = new AnnotationSet(new Integer(11), "Texas Gold Annotations",
047                "Annotations extracted from the Texas UTCS corpus.");
048    
049        private static int annotationID = 0;
050    
051        private String[] corpusFileList;
052    
053        private int currentFileIndex;
054    
055        private File corpusDirectory;
056    
057        private static GenericDocument nextTitleDocument;
058    
059        private static GenericDocument nextAbstractDocument;
060    
061        private boolean returnedTitleDocument = false;
062    
063        private boolean returnedAbstractDocument = false;
064    
065        private static List<TextAnnotation> proteinsInCurrentDocument;
066    
067        @Override
068        public boolean hasNext() {
069            /* if we have not yet returned the abstract document, then return true */
070            if (returnedTitleDocument && !returnedAbstractDocument) {
071                return true;
072            } else {
073                /* we have already returned the abstract document, so check to see if there are new title and abstract documents to return */
074                try {
075                    return hasNextDocument();
076                } catch (IOException e) {
077                    error("Error while parsing the Texas corpus directory.");
078                    e.printStackTrace();
079                    return false;
080                }
081            }
082        }
083    
084        @Override
085        public GenericDocument next() {
086            if (!returnedTitleDocument) {
087                returnedTitleDocument = true;
088                return nextTitleDocument;
089            } else {
090                returnedAbstractDocument = true;
091                return nextAbstractDocument;
092            }
093        }
094    
095        /**
096         * Initialize a new parser for the texas corpus.
097         * 
098         * @param corpusDirectory
099         */
100        public TexasCorpusParser(String corpusDirectory) {
101            File dir = new File(corpusDirectory);
102            if (dir.isDirectory()) {
103                this.corpusFileList = dir.list();
104            } else {
105                error("Input parameter: '" + corpusDirectory + "' must be a directory. Please adjust and try again");
106                System.exit(-1);
107            }
108            this.corpusDirectory = new File(corpusDirectory);
109            this.currentFileIndex = 0;
110        }
111    
112        /**
113         * Check to see if there is another document to be parsed. This is used by the Iterator methods
114         * 
115         * @return
116         * @throws IOException
117         */
118        private boolean hasNextDocument() throws IOException {
119            /* check for completion */
120            if (currentFileIndex != corpusFileList.length) {
121                String currentFilePath = corpusDirectory.getAbsolutePath() + "/" + corpusFileList[currentFileIndex++];
122                returnedAbstractDocument = false;
123                returnedTitleDocument = false;
124                File f = new File(currentFilePath);
125                /* ignore the corpus-list and tag-list files */
126                if (f.isFile()) {
127                    if (!(f.getName().equals("corpus-list") || f.getName().equals("tag-list"))) {
128                        /* process each file */
129                        String documentID = f.getName().substring(8);
130                        BufferedReader br = GenericTextUtils.openReader(currentFilePath);
131                        TexasCorpusParser.parseTexasCorpusFile(documentID, br);
132                        br.close();
133                        return true;
134                    } else {
135                        /* we have come across either the corpus-list or tag-list files.. so check for next document again */
136                        return hasNextDocument();
137                    }
138                } else {
139                    /*
140                     * do nothing.. i.e. don't recurse into any directories within the corpusDirectory, but we should check for other documents.. this
141                     * case will only occur if the user has added an extra directory
142                     */
143                    return hasNextDocument();
144                }
145            } else {
146                /* we have cycled through all of the corpus documents, so return false */
147                return false;
148            }
149        }
150    
151        public static List<TextAnnotation> getTextAnnotations() {
152            return allAnnotations;
153        }
154    
155        public static List<String[]> getDocuments() {
156            return allDocuments;
157        }
158    
159        /**
160         * Parse the corpus and store all annotations and documents into the annotation database
161         * 
162         * @param directory
163         * @throws IOException
164         */
165        public static void parseTexasCorpusDirectory(String directory) throws IOException {
166            allAnnotations = new ArrayList<TextAnnotation>();
167            allDocuments = new ArrayList<String[]>();
168    
169            File dir = new File(directory);
170            if (dir.isDirectory()) {
171    
172                String[] files = dir.list();
173    
174                for (String file : files) {
175                    File f = new File(dir.getAbsolutePath() + "/" + file);
176                    /* ignore the corpus-list and tag-list files */
177                    if (f.isFile()) {
178                        if (!(f.getName().equals("corpus-list") || f.getName().equals("tag-list"))) {
179                            /* process each file */
180                            String documentID = f.getName().substring(8);
181                            BufferedReader br = GenericTextUtils.openReader(dir.getAbsolutePath() + "/" + file);
182                            TexasCorpusParser.parseTexasCorpusFile(documentID, br);
183                            br.close();
184                        }
185                    }
186                }
187    
188            } else {
189                System.err.println("ERROR -- TexasCorpusParser: Input is not a directory.");
190            }
191    
192    //        TexasCorpusParser.storeOutput();
193        }
194    
195        /**
196         * This method parses the Texas corpus. TODO: This should be replaced at some point with the Iterator methods
197         * 
198         * @param documentID
199         * @param br
200         * @throws IOException
201         */
202        public static void parseTexasCorpusFile(String documentID, BufferedReader br) throws IOException {
203    
204            Pattern titlePattern = Pattern.compile("<ArticleTitle>(.*)</ArticleTitle>");
205            Pattern abstractPattern = Pattern.compile("<AbstractText>(.*)</AbstractText>");
206            Matcher m;
207    
208            String titleText = null;
209            String abstractText = null;
210    
211            String line;
212            while ((line = br.readLine()) != null) {
213                if (line.startsWith("<ArticleTitle>")) {
214                    m = titlePattern.matcher(line);
215                    if (m.find()) {
216                        titleText = m.group(1);
217                    } else {
218                        System.err.println("ERROR -- TexasCorpusParser: Title expected but not found.");
219                    }
220                } else if (line.startsWith("<AbstractText>")) {
221                    m = abstractPattern.matcher(line);
222                    if (m.find()) {
223                        abstractText = m.group(1);
224                    } else {
225                        System.err.println("ERROR -- TexasCorpusParser: Abstract expected but not found.");
226                    }
227                } else {
228    //                System.err.println("ERROR -- TexasCorpusParser: Unexpected start of line, " + documentID + ": " + line);
229                }
230            }
231    
232            /* create untagged document text */
233            String untaggedTitleText = titleText.replaceAll("<" + PROTEIN_TAG + ">", "").replaceAll("</" + PROTEIN_TAG + ">", "").replaceAll(
234                    GenericTextUtils.escapeCharacterForRegEx("<?>"), "").replaceAll(GenericTextUtils.escapeCharacterForRegEx("</?>"), "");
235            String untaggedAbstractText = abstractText.replaceAll("<" + PROTEIN_TAG + ">", "").replaceAll("</" + PROTEIN_TAG + ">", "")
236                    .replaceAll(GenericTextUtils.escapeCharacterForRegEx("<?>"), "").replaceAll(
237                            GenericTextUtils.escapeCharacterForRegEx("</?>"), "");
238    
239            String[] t = new String[2];
240            t[0] = documentID + "-t";
241            t[1] = untaggedTitleText;
242            allDocuments.add(t);
243    
244            nextTitleDocument = new GenericDocument();
245            nextTitleDocument.setDocumentText(untaggedTitleText);
246            nextTitleDocument.setDocumentID(documentID + "-t");
247    
248            String[] a = new String[2];
249            a[0] = documentID + "-a";
250            a[1] = untaggedAbstractText;
251            allDocuments.add(a);
252    
253            nextAbstractDocument = new GenericDocument();
254            nextAbstractDocument.setDocumentText(untaggedAbstractText);
255            nextAbstractDocument.setDocumentID(documentID + "-a");
256    
257            /* search the title for proteins */
258            if (DEBUG) {
259                System.err.println("DEBUG -- TexasCorpusParser: SEARCHING FOR TAGS IN TITLE: " + titleText);
260            }
261            searchTextForProteins(documentID + "-t", titleText, untaggedTitleText);
262            nextTitleDocument.setAnnotations(proteinsInCurrentDocument);
263            if (DEBUG) {
264                System.err.println("DEBUG -- TexasCorpusParser: SEARCHING FOR TAGS IN ABSTRACT: " + abstractText);
265            }
266            searchTextForProteins(documentID + "-a", abstractText, untaggedAbstractText);
267            nextAbstractDocument.setAnnotations(proteinsInCurrentDocument);
268    
269        }
270    
271        private static void searchTextForProteins(String documentID, String inputText, String untaggedInputText) {
272    
273            proteinsInCurrentDocument = new ArrayList<TextAnnotation>();
274    
275            openAnnotationStack = new Stack<TextAnnotation>();
276    
277            findNextTag(documentID, inputText, untaggedInputText, -1, 0);
278    
279        }
280    
281        private static void findNextTag(String documentID, String inputText, String untaggedInputText, int startSearchAtIndex,
282                int offsetAdjustment) {
283            String openTag = "<" + PROTEIN_TAG + ">";
284            String closeTag = "</" + PROTEIN_TAG + ">";
285    
286            if (DEBUG) {
287                System.err.println("DEBUG -- TexasCorpusParser: FIND NEXT TAG: startSearchAtIndex: " + startSearchAtIndex
288                        + "  offsetAdjustment: " + offsetAdjustment);
289            }
290    
291            int nextOpenTagIndex = inputText.indexOf(openTag, startSearchAtIndex);
292            int nextCloseTagIndex = inputText.indexOf(closeTag, startSearchAtIndex);
293            if (DEBUG) {
294                System.err.println("DEBUG -- TexasCorpusParser: nextOpenTagIndex: " + nextOpenTagIndex + "  nextCloseTagIndex: "
295                        + nextCloseTagIndex);
296            }
297    
298            boolean searchAgain = false;
299    
300            if ((nextOpenTagIndex > -1) && (nextOpenTagIndex < nextCloseTagIndex)) {
301                /* open new text annotation, set its start span, and push it onto the stack */
302                if (DEBUG) {
303                    System.err.println("DEBUG -- TexasCorpusParser: FOUND OPEN TAG");
304                }
305    
306                int spanStart = nextOpenTagIndex - offsetAdjustment;
307                int spanEnd = spanStart + 1;
308                TextAnnotation ta = createTextAnnotation(documentID, DOCUMENT_COLLECTION_ID, spanStart, spanEnd, ClassMentionTypes.PROTEIN, "",
309                        annotator, annotationSet, annotationID++);
310                openAnnotationStack.push(ta);
311                offsetAdjustment += openTag.length();
312                startSearchAtIndex = nextOpenTagIndex + openTag.length();
313                searchAgain = true;
314            } else if (nextCloseTagIndex > -1) {
315                /* pop the top text annotation on the stack, set its end span, and add the completed text annotation to the list */
316                if (DEBUG) {
317                    System.err.println("DEBUG -- TexasCorpusParser: FOUND CLOSE TAG");
318                }
319                TextAnnotation ta = openAnnotationStack.pop();
320                ta.setAnnotationSpanEnd(nextCloseTagIndex - offsetAdjustment);
321                /* store coveredText */
322                ta.setCoveredText(untaggedInputText.substring(ta.getAnnotationSpanStart(), ta.getAnnotationSpanEnd()));
323                allAnnotations.add(ta);
324                proteinsInCurrentDocument.add(ta);
325                offsetAdjustment += closeTag.length();
326                startSearchAtIndex = nextCloseTagIndex + closeTag.length();
327                searchAgain = true;
328            }
329    
330            if (searchAgain) {
331                findNextTag(documentID, inputText, untaggedInputText, startSearchAtIndex, offsetAdjustment);
332            } else {
333                if (DEBUG) {
334                    System.err.println("DEBUG -- TexasCorpusParser: NO MORE TAGS TO BE FOUND");
335                }
336            }
337    
338        }
339    
340    //    public static void storeOutput() throws IOException {
341    //        /* now store results in the database */
342    //        /* now add documents to the database */
343    //        for (String[] idTextPair : allDocuments) {
344    //            String documentID = idTextPair[0];
345    //            String documentText = idTextPair[1];
346    //
347    //            documentText = documentText.replaceAll("'", "''");
348    //
349    //            char TorA = documentID.charAt(documentID.length() - 1);
350    //
351    //            String sqlStr = "INSERT INTO TEXAS_CORPUS VALUES ('" + documentID + "', '" + Character.toUpperCase(TorA) + "', '"
352    //                    + documentText + "')";
353    //            System.out.println("SQLSTR: " + sqlStr);
354    //            Database_Util.execute(AnnotationDBProperties.CONNECTION_PROPERTIES_FILE, sqlStr);
355    //        }
356    //
357    //        String sqlldrFileName = "texas_Annotations.sqlldr";
358    //        String runLoadFileName = "loadTexasAnnotations.sh";
359    //
360    //        /* add the annotations to the database in the form of an sqlldr file */
361    //        /* create the sqlldr input file */
362    //        PrintStream sqlldrFileStream = GenericTextUtils.openPrintStream(AnnotationDBProperties.EXTERNAL_BASE_ANNOTATION_DIR
363    //                + "/inputFiles/" + sqlldrFileName);
364    //        AnnotationDB_Util.printTextAnnotationListToSqlldrFile(sqlldrFileStream, allAnnotations, new ArrayList<String>(), true,
365    //                AnnotationDBProperties.CONNECTION_PROPERTIES_FILE, false);
366    //        sqlldrFileStream.close();
367    //
368    //        /* add the parsed gene annotations to the database */
369    //        AnnotationDB_Util.loadAnnotationsIntoDB(AnnotationDBProperties.EXTERNAL_BASE_ANNOTATION_DIR,
370    //                AnnotationDBProperties.LOCAL_BASE_ANNOTATION_DIR, sqlldrFileName, AnnotationDBProperties.SQLLDR_CONTROL_FILE_TEMPLATE,
371    //                runLoadFileName, AnnotationDBProperties.ANNOTATION_DB_STATUS_FLAG_FILE, true);
372    //
373    //    }
374    
375        public void close() throws IOException {
376        }
377    
378        /**
379         * args[0] = texas corpus directory
380         * 
381         * @param args
382         */
383        public final static void main(String[] args) {
384            try {
385                TexasCorpusParser.parseTexasCorpusDirectory(args[0]);
386            } catch (IOException e) {
387                e.printStackTrace();
388            }
389        }
390    
391    }
```
